# Soft-Templated Sol–Gel Synthesis of Mesoporous Perovskite-Type Multicomponent Metal Oxide (Bi_0.2_Na_0.2_K_0.2_La_0.2_Sr_0.2_)TiO_3_ and Its Enhanced Photocatalytic Activity

**DOI:** 10.1021/acsomega.5c12465

**Published:** 2026-01-05

**Authors:** Hanzo Tsubota, Kazuki Inoue, Wai Kian Tan, Hiroyuki Muto, Atsunori Matsuda, Andrei Jitianu, Go Kawamura

**Affiliations:** † Department of Electrical and Electronic Information Engineering, 13129 Toyohashi University of Technology, 1-1 Hibarigaoka, Tempaku-cho, Toyohashi, Aichi 441-8580, Japan; ‡ Institute for Research on Next Generation Semiconductor and Sensing Science, Toyohashi University of Technology, 1-1 Hibarigaoka, Tempaku-cho, Toyohashi, Aichi 441-8580, Japan; § Institute of Liberal Arts and Sciences, Toyohashi University of Technology, 1-1 Hibarigaoka, Tempaku-cho, Toyohashi, Aichi 441-8580, Japan; ∥ Department of Chemistry, Lehman College-CUNY, Davis Hall, 250 Bedford Park Boulevard West, Bronx, New York 10468, United States; ⊥ Ph.D. Program in Chemistry and Biochemistry, The Graduate Center of the City University of New York, 365 Fifth Avenue, New York, New York 10016, United States

## Abstract

The high calcination temperature required for the crystallization of multicomponent metal oxides is a challenge because this treatment significantly reduces their specific surface area while increasing the crystallite size, thereby limiting their photocatalytic performances. In this study, we have addressed this issue for a multicomponent perovskite photocatalyst, (Bi_0.2_Na_0.2_K_0.2_La_0.2_Sr_0.2_)­TiO_3_ (BNKLST), by employing a soft-templating sol–gel method using Pluronic F-127. The synthesis process was optimized by adjusting the solution’s pH to ensure uniform micelle self-assembly and by determining the optimum calcination temperature for the pore formation. This approach enabled the successful low-temperature synthesis of BNKLST, which retained a high specific surface area of 50.4 m^2^ g^–1^ at 400 °C. The resulting catalyst exhibited excellent photocatalytic activity, degrading methylene blue under UV irradiation (250–450 nm) at a rate more than six times faster than that obtained using commercial SrTiO_3_. These findings demonstrate that enhancing the specific surface area of multicomponent metal oxides through a soft-templating approach is an effective strategy in photocatalyst design.

## Graphical abstract

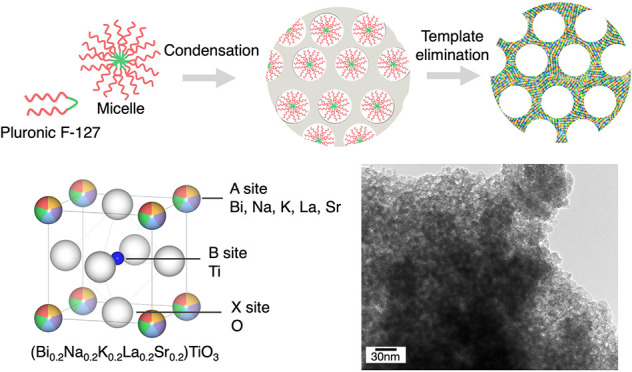

## 1 Introduction

Semiconductor photocatalysts that convert solar energy into chemical energy are attracting intense interest as a key technology for environmental protection and remediation. The potential applications of these materials range from hydrogen evolution and CO_2_ reduction to the removal of pollutants from water and air.^1^ Yet widely studied oxide photocatalysts such as TiO_2_ and ZnO still exhibit low solar-to-chemical conversion efficiencies, spurring the pursuit for new, more effective catalysts.^2,3^ Among these, multicomponent metal oxides, often referred to as high-entropy oxides (HEOs), which incorporate multiple elements, have garnered significant attention as next-generation photocatalysts due to their unique crystal structures, tunable band structures, abundant active sites, and high stability.^4–7^ HEOs are characterized by structural innovation through the introduction of multiple cations into the lattice. In the last years the research reports on their photocatalytic applications have been increasing, although the field is still in its early stages.^8,9^ However, many of these multicomponent metal oxides, particularly when prepared by conventional solid-state reaction from oxide powders, require high temperatures of calcination (typically around 1000 °C) to form homogeneous solid solutions.^10–12^ For example, the synthesis of a dual-phase (monoclinic and orthorhombic) TiZrNbHfTaO_11_ HEO for CO_2_ photoreduction has been reported to involve a high-pressure torsion step followed by a final oxidation at 1100 °C.^12^ Such high-temperature treatments promote grain growth and sintering, resulting in a significant decrease in the specific surface area. This reduction of the surface area, which provides active sites for photocatalytic reactions, has been a limiting factor for their broader application.^13^ Since photocatalytic reactions predominantly occur on the surface of the catalyst, a large specific surface area is a crucial factor for achieving high photocatalytic activity.

The sol–gel method using soft templates, such as surfactants, is widely recognized as an effective technique to increase the specific surface area of metal oxides. By using this approach, micellar self-assembly templates nanoscale pores are formed, thereby creating a mesostructure high specific surface area.^14,15^ For instance, the use of the block copolymer F-127 as a soft template in a sol–gel synthesis has been shown to produce mesoporous honeycomb iron titanate with a large specific surface area of 132.74 m^2^ g^–1^.^16^ Nevertheless, only a few studies have applied soft templating to multicomponent oxides containing five or more elements for photocatalysis.^17,18^ Achieving a uniform reaction that synchronizes the formation of a multication network in the liquid phase with micellar self-assembly is extremely challenging and demands meticulous optimization of reaction parameters for each system.^19,20^ Although low calcination temperatures are effective for creating high specific surface areas via soft templating, they often result in materials with poor crystallinity.^21–23^ Establishing well-controlled processing parameters can overcome these barriers and unlock the full potential of multicomponent metal oxides by maximizing their otherwise limited specific surface area.

Characterized by the general formula ABO_3_, perovskites feature an A-site typically occupied by a large alkali, alkaline earth, or rare-earth cation and a B-site occupied by a transition metal cation. The inherent flexibility of these sites to host a diverse range of elements has facilitated their expansion into the field of HEOs.^24^ A significant challenge, however, is that the compositional complexity of these high-entropy perovskites necessitates even higher synthesis temperatures (1200–1600 °C) compared to HEOs with other crystal structures.^25,26^ This requirement, in turn, has severely limited their application in photocatalysis, where a high specific surface area is crucial for the performance. On the other hand, their single-component counterparts are well established as photocatalysts. Strontium titanate (SrTiO_3_), in particular, is a benchmark material, exhibiting a high dielectric constant, favorable electrical conductivity, and band edge positions suitable for overall water splitting (OWS). Furthermore, its photocatalytic activity and light absorption can be enhanced through doping with metal ions like Al^3+^.^27^ However, reports on the photocatalytic applications of SrTiO_3_-based multicomponent perovskites are scarce.^28–30^ To address this gap, we aim to obtain (Bi_0.2_Na_0.2_K_0.2_La_0.2_Sr_0.2_)­TiO_3_ (BNKLST) nanostructures with enhanced specific surface areas by systematically varying two key processing parameters: The pH of the sol and the calcination temperature. To the best of our knowledge, this composition has not been previously reported as a photocatalyst. The resulting samples were then subjected to comprehensive characterization of their crystal structure, morphology, surface chemical states, and optical absorption properties, with the aim of clarifying how these features influence photocatalytic activity.

## 2 Experimental Methods

### 2.1 Materials

For the synthesis of BNKLST, titanium­(IV) oxyacetylacetonate (C_10_H_14_O_5_Ti, TiO­(acac)_2_) (90%, Sigma-Aldrich), bismuth­(III) nitrate pentahydrate (Bi­(NO_3_)_3_·5H_2_O) (99.0%, Wako), sodium nitrate (NaNO_3_) (Wako, 99.0%), potassium nitrate (KNO_3_) (99.0%, Wako), lanthanum­(III) nitrate hexahydrate (La­(NO_3_)_3_·6H_2_O) (99.0%, Wako), strontium nitrate (Sr­(NO_3_)_2_) (99.0%, Wako), Pluronic F-127 block copolymer ((C_2_H_4_O)_100_(C_3_H_6_O)_65_(C_2_H_4_O)_100_) (Sigma-Aldrich), hydrochloric acid (HCl) (35.0- 37.0%, Wako), ethanol (C_2_H_5_OH) (99.5%, Wako) were used. Methylene blue (C_16_H_18_N_3_SCl·3H_2_O) (Wako) was used for photocatalytic dye degradation measurements. All aqueous solutions were prepared with deionized water. All chemicals were used without further purification.

### 2.2 Sample Preparation

BNKLST with a mesoporous structure was synthesized using an adapted method originally employed for the preparation of ZnTiO_3_ reported by Li et al.^31^ Here, we prepared BNKLST via a sol–gel method using metal nitrates and TiO­(acac)_2_ as the starting materials. Initially, 25 mmol of TiO­(acac)_2_ was dissolved in 40 mL of ethanol, followed by the addition of 1.6 g of F-127. This mixture was stirred for 1 h. Separately, 5 mmol each of various metal nitrates were added to 3 mL of deionized water, and 400 μL of hydrochloric acid (35–37%) was introduced to facilitate the dissolution of Bi­(III) nitrate. After the complete dissolution of the metal nitrates was confirmed, this solution was gradually added to the ethanol solution of TiO­(acac)_2_. Initially, samples were prepared without pH adjustment (pH 1.0), while others were prepared with their pH adjusted to 3.7, 6.5, 9.0, and 11 using aqueous ammonia (28%). The mixed solution was stirred for 5 h, followed by aging at 40 °C for 2 days to obtain the calcination precursor. The precursor was then dried at 100 °C for 24 h and calcined at 400 °C. For comparison, BNKLST samples without F-127 were also prepared under conditions of no pH adjustment (pH 1.0) and at an adjusted pH of 9.0, and then calcined at 400 °C. Furthermore, to investigate the effect of calcination temperature, samples with an initial pH of 9.0 were calcined at 400, 500, 600, and 1000 °C.

### 2.3 Structural Characterization

Specific Surface area­(*S*_BET_) and porosity were calculated by Brunauer–Emmett–Teller (BET) and Barrett–Joyner–Halenda (BJH) analyses of N_2_ adsorption/desorption isotherms obtained from a Tristar II instrument (Micromeritics, USA) at 77 K, and all samples were outgassed in a vacuum at 110 °C for 2 h. Crystal structure was analyzed by X-ray diffraction (XRD) using an Ultima IV diffractometer (Rigaku, Japan) with Cu Kα radiation. The crystalline size was calculated using the Scherrer eq (eq 1), where “*D*” is the crystallite size (nm), “θ” is the diffraction angle (rad),“*K*” is the Scherrer constant, “λ” is the wavelength of X-rays (*Cu K*α: 1.5418 Å), and “β” is the full width at half-maximum (FWHM).^32^ The thermal behavior of the BNKLST precursors was analyzed by thermogravimetric and differential thermal analysis (TG-DTA) using a Thermo plus EVO2 instrument (Rigaku, Japan) in air from 25 to 1000 °C at a heating rate of 2 °C min^–1^. Detailed morphology was explored using a transmission electron microscope (TEM) with a field-emission gun operating at 200 kV (JEM-2100F, JEOL, Japan). The elemental analyses for composite materials were carried out using scanning TEM (STEM)-EDX (JEM-2100F, and JED-2300T, JEOL, Japan). Elemental composition and chemical state were analyzed by X-ray photoelectron spectroscopy (XPS) using a PHI Quantera SXM-CI spectrometer (ULVAC PHI, Japan) with Al Kα radiation. The spectra were analyzed with MultiPak software (ULVAC-PHI). Optical absorption properties were evaluated by ultraviolet–visible diffuse reflectance spectroscopy (UV–vis DRS) using a V-670 spectrophotometer (JASCO, Japan) over a spectral range of 300–800 nm with a resolution of 0.2 nm.

1 $$D=\frac{K\lambda }{\beta \cos\theta }$$

### 2.4 Photocatalytic Activity

Photocatalytic experiments were performed at room temperature under atmospheric pressure. The prepared sample (50 mg) of the photocatalyst was dispersed in 50 mL of methylene blue (MB) solution (7 mg/L) and stirred in the dark for 60 minutes in a beaker to reach adsorption equilibrium. The mixture was then irradiated with a UV lamp (Spot Cure SP-7, 250–450 nm, 20 mW/cm^2^) for 120 minutes. At 20 min intervals, 1.5 mL samples were taken, and the photocatalyst particles were removed by centrifugation at 12,000 rpm for 30 s. MB concentration was measured using a V-670 spectrophotometer (JASCO, Japan) with deionized water as a reference. Changes in MB concentration were evaluated based on the integrated absorbance area within the wavelength range of 550–750 nm with a resolution of 0.2 nm. The pseudo-first-order kinetic of photodegradation rate of MB was determined using the Langmiur–Hinshelwood kinetic model­(eq 2), where, *C*_0_ is the dye concentration at initial, *C* is the dye concentration at time *t* (min), *k* is the pseudo-first-order rate constant, and *t* is the irradiation time.^33^

2 $$\ln\frac{C}{C_{0}}=-\mathrm{kt}$$

### 2.5 Configurational Entropy

The configurational entropy (*S*) of the perovskite oxide was calculated according to eq 3. In this equation, *x*_*a*_, *x*_*b*_, and *x*_*c*_ represent the mole fractions of the ions in the A, B, and O sites, respectively. *R* is the ideal gas constant, 8.314 J/K mol, and *n*_*a*_, *n*_*b*_, and *n*_*c*_ are the number of elements.^34^ The calculation was based on the stoichiometric formula of BNKLST, neglecting the potential oxygen deficiency, which resulted in an *S* value of 1.61*R*. As this value for *S* is greater than 1.50 *R*, the system can be classified as a high-entropy material.^35^

3 $$S=-R\left(\sum_{a=1}^{n_{a}}x_{a}\ln x_{a}+\sum_{b=1}^{n_{b}}x_{b}\ln x_{b}+3\sum_{c=1}^{n_{c}}x_{c}\ln x_{c}\right)$$

## 3 Results and Discussion

### 3.1 Specific Surface Area and Porosity

Nitrogen adsorption–desorption measurements were performed to evaluate the specific surface area and pore structure of the BNKLST powders. To identify the influence of the pH of the synthesis, a series of samples were prepared with and without templating using agent F-127, and then calcined at 400 °C. As displayed in Figure 1a, type IV isotherms, characteristic of mesoporous materials, were identified for the studied samples.^36^ Notably, the specimens prepared without F-127 also show a type IV isotherm, suggesting that intrinsic mesopores are generated by the decomposition of the organic acetylacetonate ligand, which remained entrapped in the samples. As summarized in Table 1, the BET surface areas of the F-127-free samples (pH 3.7 and pH 9.0) are limited to 3.18 and 12.7 m^2^ g^–1^, respectively. In sharp contrast, the incorporation of F-127 enhances the surface area; the specimen synthesized at pH 9.0 reaches a maximum of 50.4 m^2^ g^–1^ with a pore volume of 0.16 cm^3^ g^–1^. This maximum surface area, achieved at pH 9.0, is attributed to the stable formation of F-127 micelles, which act as effective templates for an ordered mesoporous network. At lower (pH 1.0–6.5) or higher (pH 11) pH, micelle stability deteriorates, leading to smaller surface areas. The pore-size distribution presented in Figure 1b validates that the F-127-templated samples possess a narrow mesopore distribution at 5–15 nm.

**Table 1:** BET Surface Area and Porosity Measurements of BNKLST Synthesized at Various pH Values with and Without Pluronic F-127, and Calcined at 400 °C

sample	pH	*S*_BET_ (m^2^ g^–1^)	pore volume (cm^3^ g^–1^)
BNKLST + F-127	1.0	35.2	0.14
	3.7	25.5	0.076
	6.5	25.4	0.070
	9.0	50.4	0.16
	11	35.4	0.11
BNKLST	3.7	3.18	0.041
	9.0	12.7	0.079

**Figure 1:**
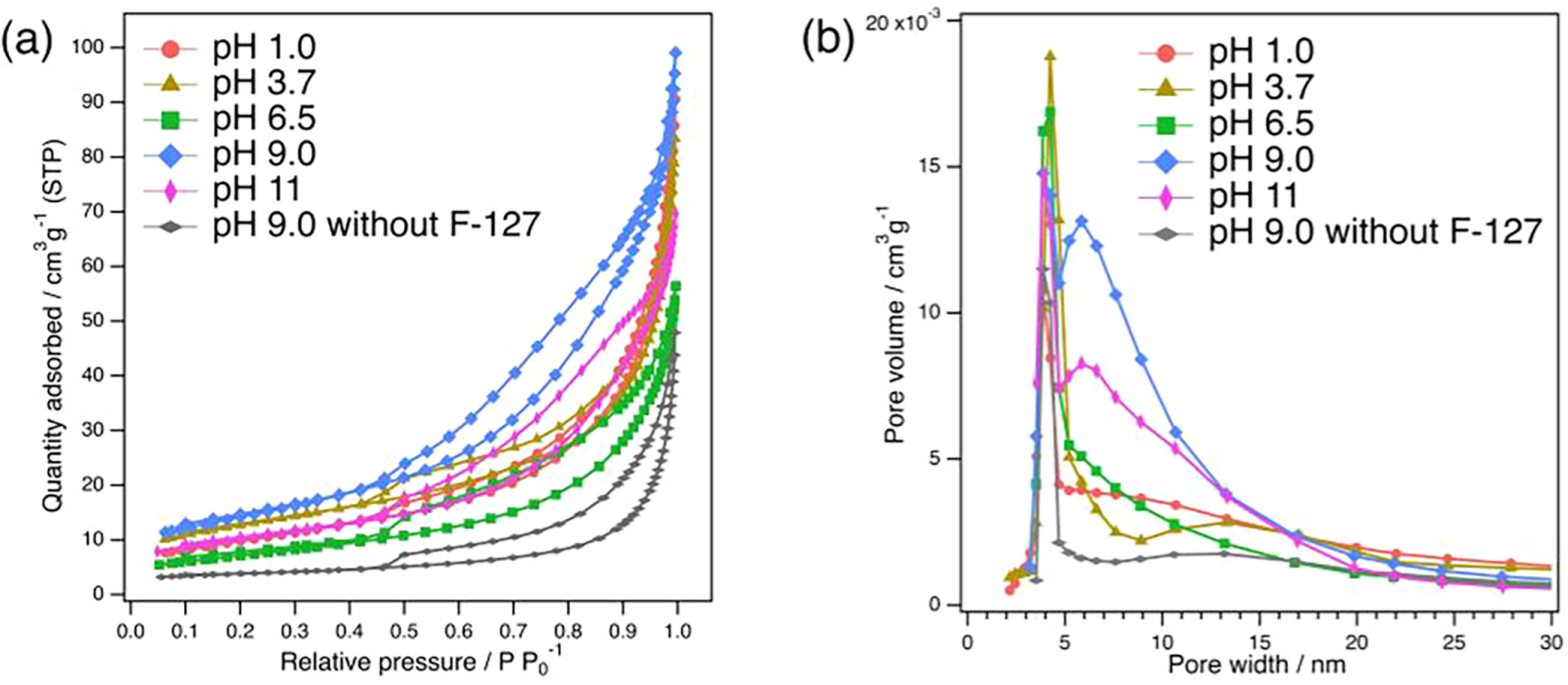
(a) N_2_ adsorption–desorption isotherms and (b) pore-size distributions of BNKLST synthesized at various pH values with and without Pluronic F-127, and calcined at 400 °C.

Based on this, for the sample prepared at pH 9.0, which exhibited the largest specific surface area upon calcination at 400 °C, the effect of calcination temperature on these structural properties was evaluated in detail (Figure 2 and Table 2). Raising the calcination temperature from 400 to 1000 °C results in a monotonic decrease of the BET surface area, as presented in Table 2. Although type IV hysteresis is retained up to 600 °C, the loop collapses at 1000 °C, and the isotherm changes to Type III. The pore volume shrinks to 0.084 cm^3^ g^–1^, indicating extensive pore closure owing to crystallite growth and sintering. (Figure 2a). Consistently, Figure 2b reveals a slight coarsening of the pore-size distribution with increasing temperature, reflecting the loss or merging of smaller pores to form larger pores. From these results, it was suggested that F-127 templating at pH ≈ 9 followed by low-temperature calcination at 400 °C is necessary for obtaining BNKLST with a developed mesoporous framework and a large specific surface area.

**Table 2:** BET Surface Area and Porosity Measurements of BNKLST Synthesized at pH 9.0 with Pluronic F-127 and Calcined at Different Temperatures

sample	calcination temperature (°C)	*S*_BET_ (m^2^ g^–1^)	pore volume (cm^3^ g^–1^)
BNKLST + F-127	400	50.4	0.16
	500	30.8	0.14
	600	28.3	0.15
	1000	8.99	0.084

**Figure 2:**
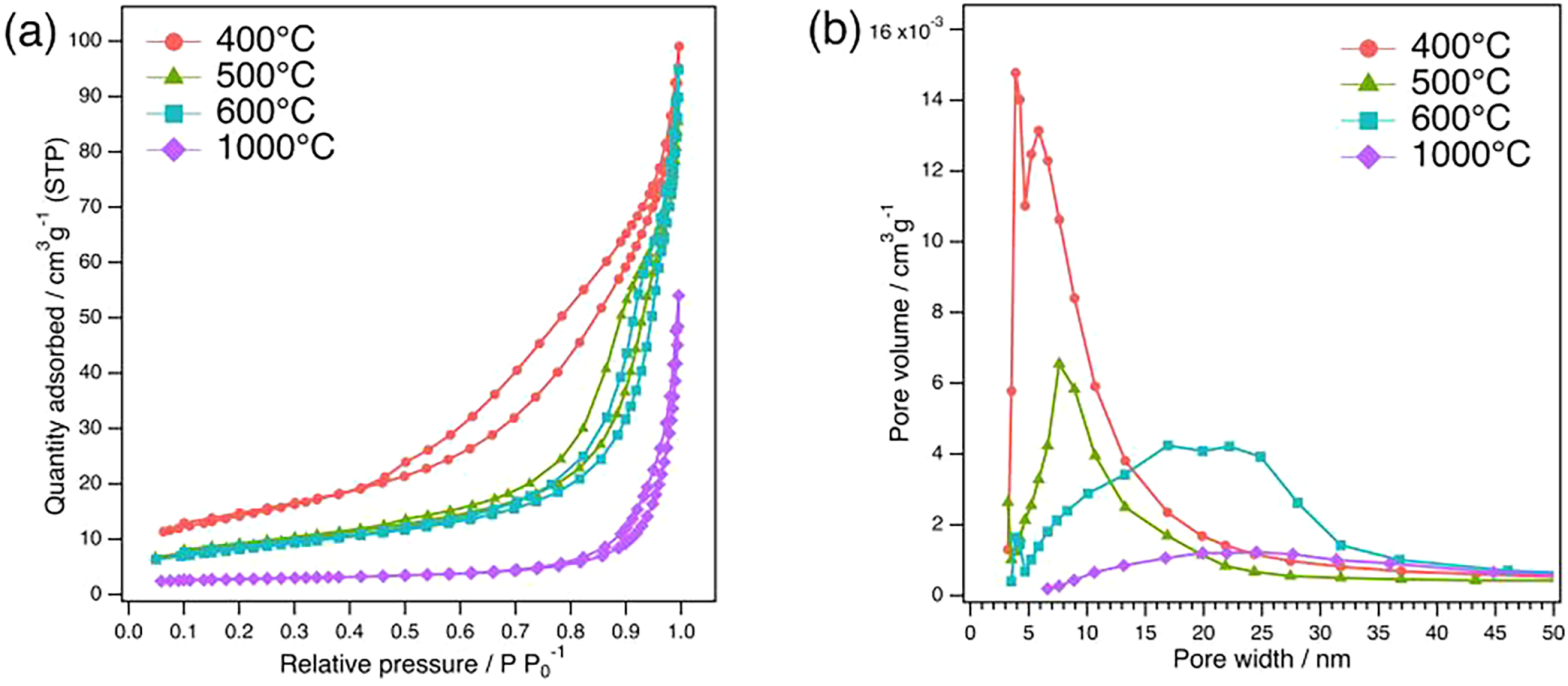
(a) The N_2_ adsorption–desorption isotherms and (b) pore-size distributions of BNKLST synthesized at pH 9.0 with Pluronic F-127 and calcined at different temperatures.

### 3.2 Crystal Structure Analysis

The relationship between the changes in specific surface area and pore structure and the material’s crystal structure and phase formation was analyzed. Phase identification was performed by comparing the obtained XRD patterns with reference data from the Inorganic Crystal Structure Database (ICSD).^37^ Figure 3 presents the XRD patterns of BNKLST samples synthesized with F-127 at pH 9.0 and subsequently calcined at different temperatures as outlined. The XRD pattern of the sample calcined at 400 °C revealed a mixed-phase composition, consisting of a poorly crystalline perovskite phase (SrTiO_3_, ICSD No. 23076), as indicated by its broad, low-intensity peaks, and a secondary phase belonging to the aragonite group (SrCO_3_, ICSD No. 15195). This incomplete crystallization is likely due to a delay in phase formation caused by the presence of the F-127 template and unreacted organic moieties, which are not fully decomposed at this low calcination temperature. As the calcination temperature was increased to 500 °C and subsequently to 600 °C, the diffraction peaks corresponding to the perovskite phase tended to become sharper and more intense. This indicates that the crystallization of BNKLST progressed with an increase in the calcination temperature. On the other hand, the intensity of the peak that was assigned to the aragonite-group phase became stronger with an increase in temperature up to 600 °C, indicating an increase of the crystallite size of the aragonite-group phase. Moreover, in the XRD pattern of the sample calcined at 1000 °C, peaks originating from the phase that belongs to the aragonite group almost completely disappeared, and only sharp, intense diffraction peaks characteristic of the perovskite structure were clearly identified. This result strongly indicates that high-temperature calcination at 1000 °C led to the decomposition of the phase that belongs to the aragonite group and/or its incorporation into the perovskite phase formation, alongside the complete removal of the residual organics, thereby yielding highly crystalline, single-phase perovskite BNKLST. A close relationship is expected between the crystallite size obtained from XRD data and the aforementioned BET specific surface area; the combined effect of calcination temperature on the crystallite size and specific surface area of BNKLST is illustrated in Figure 4. The crystallite size of the perovskite phase, calculated from the (110) diffraction peak at 2θ ≈ 32° using the Scherrer eq 1, tended to increase as the calcination temperature increased from 400 to 1000 °C. Conversely, the specific surface area measured by the BET method, as previously shown in Table 2 and Figure 2, decreased with increasing calcination temperature. These results demonstrate a clear inverse correlation between crystallite size and specific surface area. That is, while calcination at higher temperatures enhances crystallinity and promotes particle size growth, it also facilitates particle agglomeration by sintering, leading to the collapse of porous structures and densification, which consequently results in a reduction in specific surface area.

**Figure 3:**
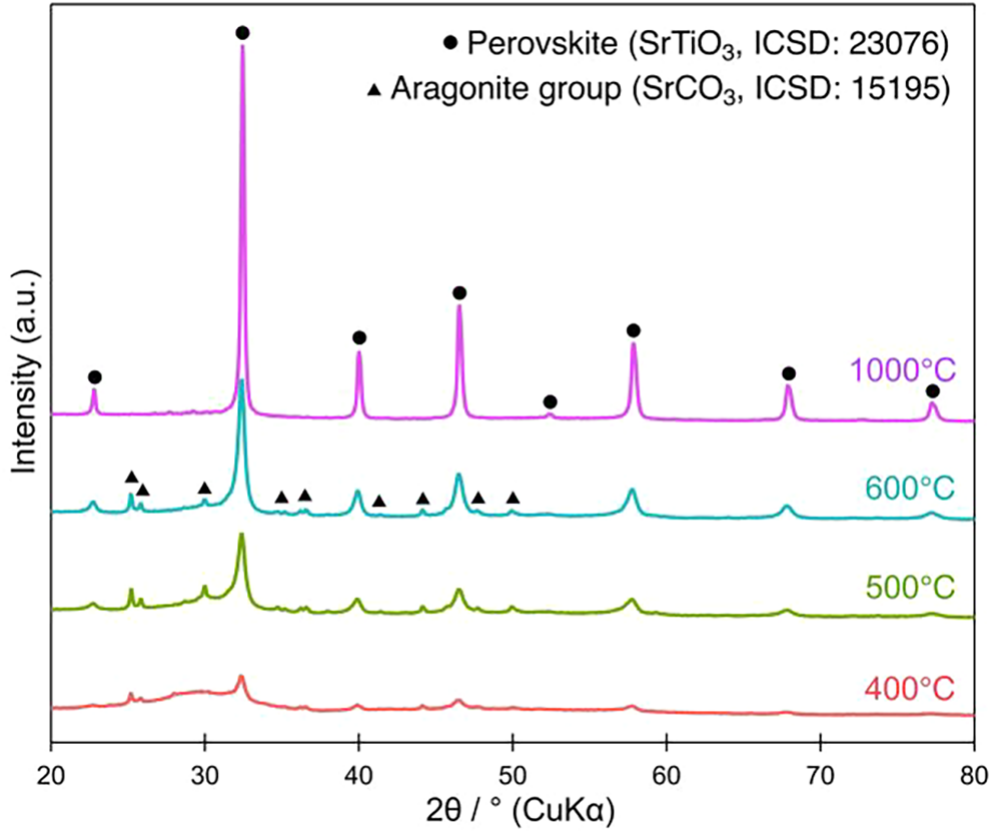
XRD patterns of BNKLST synthesized at pH 9.0 with Pluronic F-127 and calcined at different temperatures.

**Figure 4:**
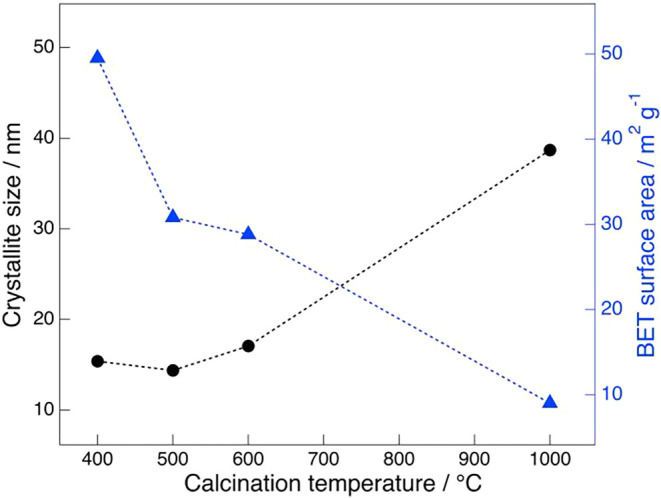
Relationship between crystallite size and BET surface area by calcination temperature of BNKLST with F-127 synthesized at pH 9.0.

### 3.3 Thermal Analysis

TG-DTA was performed on the BNKLST precursor synthesized with F-127 under pH 9.0 conditions to investigate in detail the temperature regions where structural changes and phase transitions occur (Figure 5). The TG curve reveals a total weight loss of approximately 75% upon heating to 1000 °C, which indicates the removal of volatile components and decomposition of organic species. This weight loss can be categorized into four distinct steps. The first weight loss is observed below 150 °C. This stage is characterized on the DTA curve by a small exothermic peak around 110 °C, followed by an endothermic peak. The exothermic peak is attributed to polymerization reactions associated with the formation of the inorganic polymeric network.^38^ The subsequent endothermic peak primarily corresponds to the evaporation of physically adsorbed water. The second major weight loss step was observed between 150 °C and up to around 550 °C, with the most significant thermal effects being concluded by 400 °C. This range encompasses several exothermic events. Specifically, a pronounced exothermic peak is noted at 182 °C, which is attributed to the beginning of decomposition of the F-127.^39^ Subsequently, another exothermic peak is observed at 237 °C, originating from TiO­(acac)_2_, followed by a further exothermic effect at 338 °C, which suggests the combustion of residual carbon species from F-127.^40^ A distinct endothermic peak is observed at approximately 928 °C, which is preceded by a minor mass gain from oxidation at approximately 910 °C and followed by a final slight weight loss up to 1000 °C. Based on the XRD results, which show a transition from a mixed perovskite phase/aragonite-group phase at lower temperatures to a single-phase perovskite structure at 1000 °C, this endothermic effect is interpreted as the structural transformation associated with the complete decomposition of the phase, which belongs to the aragonite-group and its incorporation into the perovskite ˙lattice.

**Figure 5:**
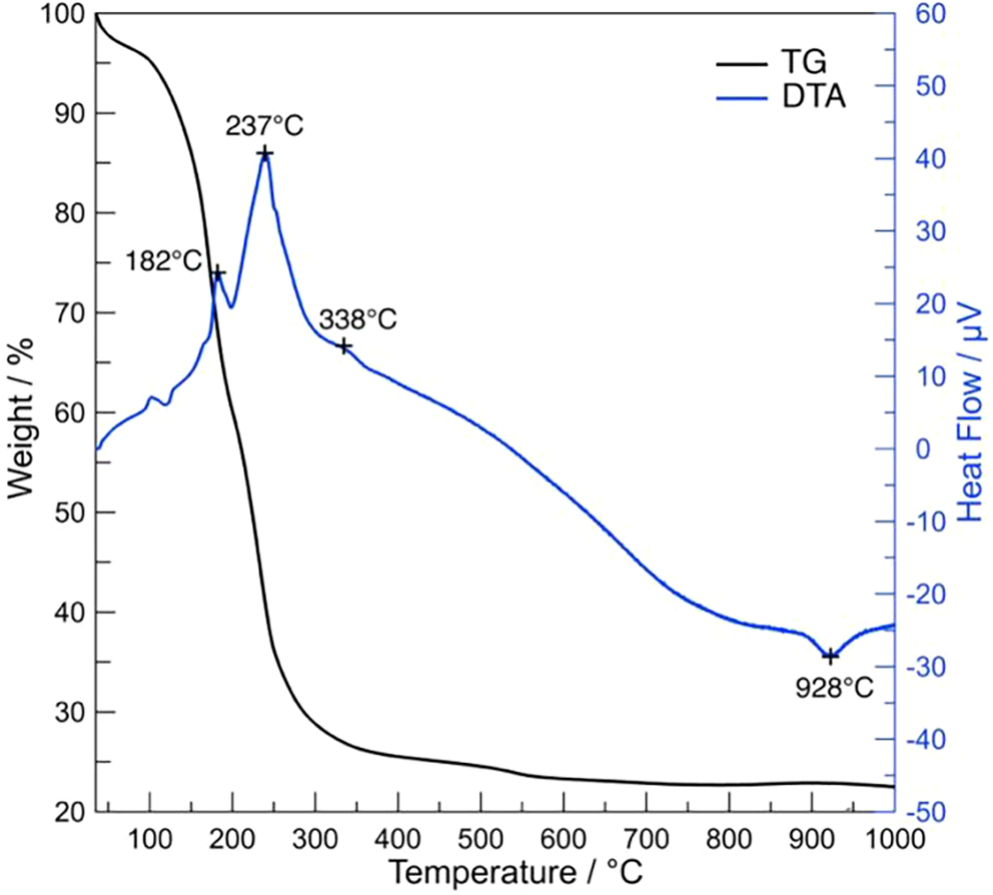
TG-DTA curves of BNKLST with F-127 synthesized at pH 9.0 after drying at 100 °C.

### 3.4 Microstructural Characterization and Elemental Mapping

The nanoscale morphology and elemental mapping of these materials were observed using TEM and STEM-EDX. First, the microstructure of BNKLST samples synthesized at pH 9.0 and calcined at 400 °C was compared, with and without the addition of the surfactant F-127. As shown in Figure 6a, the sample prepared with F-127 exhibits a distinct porous morphology characterized by uniformly dispersed nanocrystals with numerous nanoscale voids. The feature is indicative of the successful formation of mesoporous structures templated by F-127 micelles during the sol–gel process.^41^ In contrast, the sample synthesized without F-127 (Figure 6b) presents a dense and aggregated structure lacking any visible porosity. These results suggest that the incorporation of F-127 contributes to the formation of mesoporous structures and implies that F-127 may function as a soft template in controlling the nanostructure of the final product. As shown in Figure 6c, when the F-127-templated BNKLST sample was calcined at 1000 °C, the porous features observed at lower temperatures (Figure 6a) disappeared almost completely. The microstructure became denser, and the particles appeared to grow significantly, exhibiting well-defined boundaries and grain-like shapes. This transformation is attributed to the sintering of the particles and grain growth at high temperatures, which leads to the collapse of the mesoporous framework originally introduced by F-127. These morphological changes are consistent with the observed reduction of the surface area and the pore volume, and they underscore the necessity of optimizing the calcination temperature to preserve the mesostructural integrity obtained during the synthesis.

**Figure 6:**
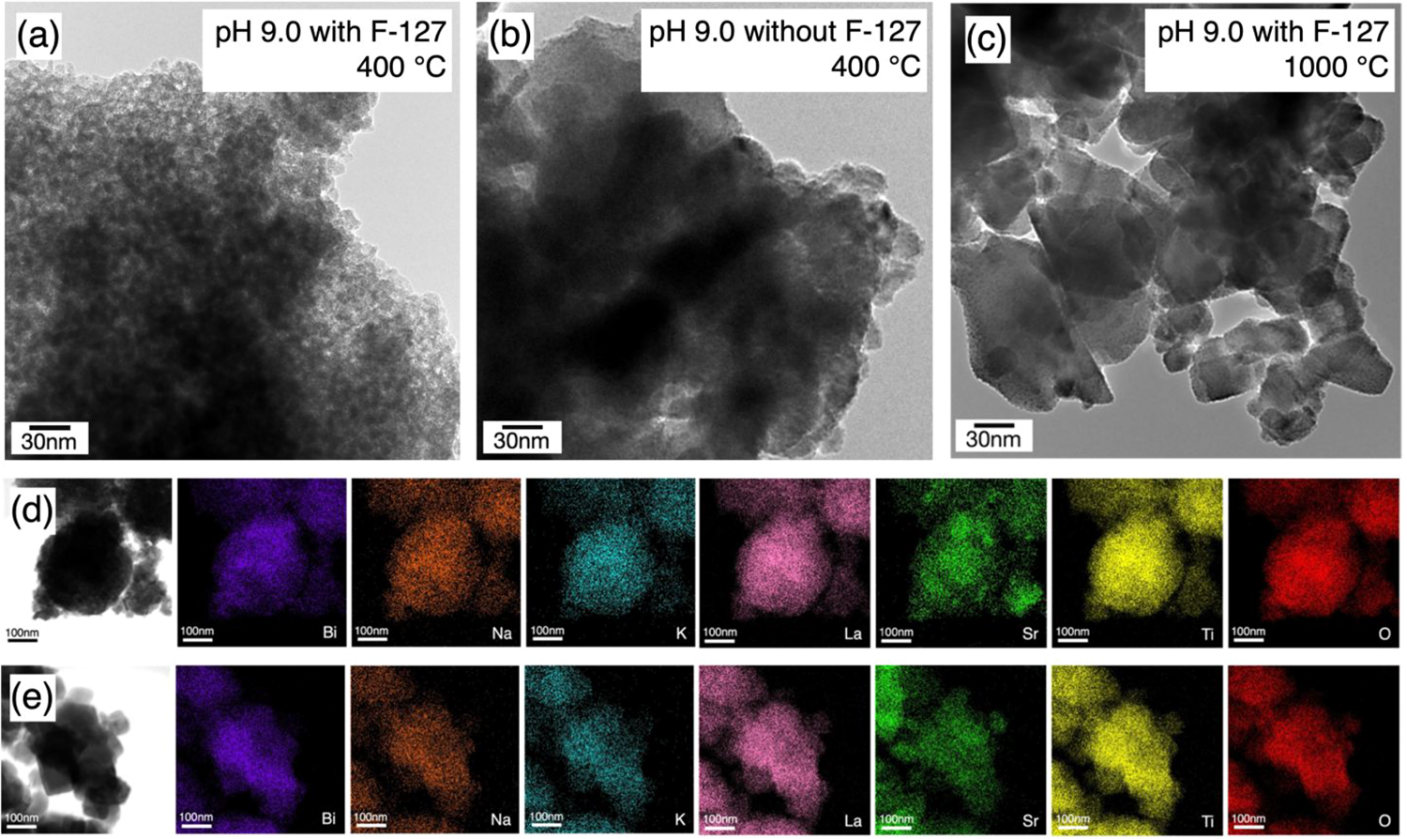
TEM images of BNKLST synthesized at pH 9.0 (a) with F-127 (b) without F-127, calcined at 400 °C, and (c) with F-127 calcined at 1000 °C. STEM-EDX elemental mappings of BNKLST with F-127 synthesized at pH 9.0, calcined at (d) 400 and (e) 1000 °C.

The elemental homogeneity in these samples calcined at 400 °C and 1000 °C was evaluated by STEM-EDX. The elemental mapping results for the sample calcined at 400 °C, presented in Figure 6d, show that while the constituent elements bismuth, sodium, potassium, lanthanum, titanium, and oxygen are distributed throughout the particles, some localized aggregation in the distribution of strontium was observed. This nonuniform distribution of strontium is considered to be related to the presence of the aragonite-group phase, which was identified during the XRD analysis (Figure 3) for the sample calcined at 400 °C. This suggests that at the lower calcination temperature, a portion of the strontium was not incorporated into the perovskite structure and existed separately as a carbonate. On the other hand, the elemental mapping results for the sample calcined at 1000 °C, shown in Figure 6e, confirm that all constituent elements were relatively uniformly distributed throughout the observed particle regions. Notably, the random distribution of strontium observed for the 400 °C sample was resolved for the sample calcined at 1000 °C. This strongly suggests that all elements were homogeneously incorporated into the perovskite lattice. This uniform elemental distribution aligns well with the XRD analysis results, which indicated that the sample calcined at 1000 °C consisted of a single perovskite phase. This demonstrates that high-temperature calcination promoted the decomposition of the aragonite-group phase and the diffusion of each constituent element, contributing to the formation of a homogeneous perovskite structure. With a configurational entropy of 1.61*R*, this final structure is classified as a HEO.

### 3.5 Surface Chemical State Analysis

XPS analysis was conducted to investigate the elemental composition and oxidation states of the ions on the surface of the BNKLST samples. Figure 7 displays the XPS spectra for the sample synthesized with F-127 at pH 9.0 and calcined at 400 °C, while Figure 8 shows the spectra for the sample similarly prepared but calcined at 1000 °C. The high-resolution XPS spectra for Bi 4f, Na 1s, K 2p, La 3d, and Sr 3d confirmed that these elements were present in their typical oxidation states: Bi­(III), Na­(I), K­(I), La­(III), and Sr­(II), respectively. No significant differences were observed in the spectral shapes of these ions between the samples calcined at 400 and 1000 °C. For the Ti 2p spectra, shown in Figures 7 and 8, the main peaks observed at approximately 458.0 eV for Ti 2p_3/2_ and 464.5 eV for Ti 2p_1/2_ are attributed to the presence of Ti­(IV). The shoulders on the lower binding energy side of these main peaks, observed for both samples at approximately 457.0 and 462.8 eV, respectively, are attributed to the presence of Ti­(III). The existence of Ti­(III) is likely associated with oxygen vacancies within the perovskite structure for charge compensation.^42^ In general, multicomponent metal oxides, due to the coexistence of multiple metal cations with different ionic radii within the same lattice, tend to exhibit lattice distortion, which readily leads to the introduction of oxygen deficiency.^43^ The spectra of the O 1s could be deconvoluted into multiple components. The main component at approximately 529.5 eV is attributed to lattice oxygen (M–O–M bonds), the peak around 531.5 eV is attributed to surface hydroxyls and oxygen ions in oxygen-deficient regions, which indirectly reflect the presence of oxygen vacancies, and the component at approximately 533.2 eV corresponds to surface-adsorbed oxygen species.^44,45^ The relative intensity of this defect-related component indicates the presence of oxygen-deficient regions, which is consistent with the coexistence of Ti­(III) observed in the Ti 2p spectra. Importantly, for the sample calcined at 1000 °C, the relative intensity of this component is weaker compared with that of the sample calcined at 400 °C.

**Figure 7:**
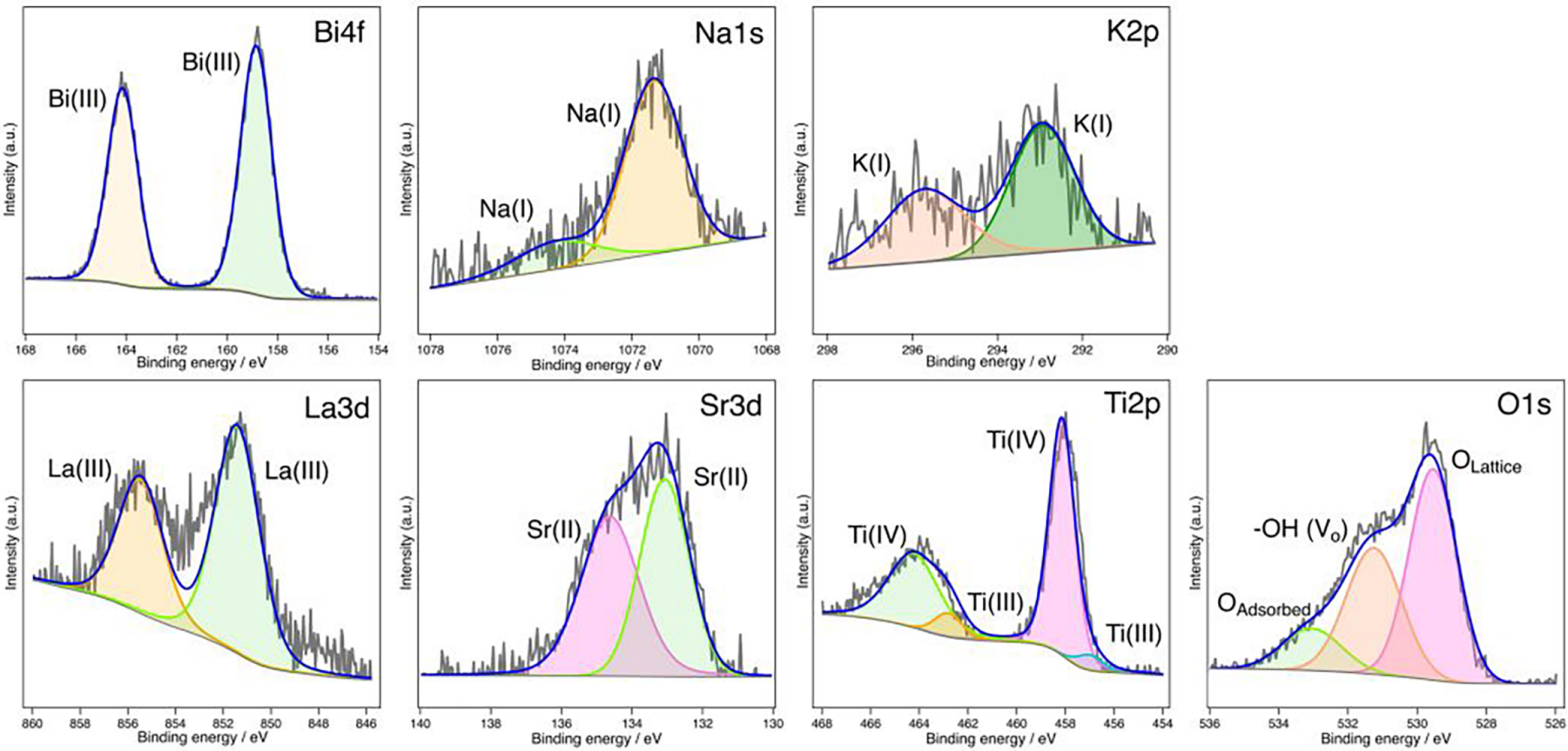
XPS spectra of BNKLST with F-127 synthesized at pH 9.0, calcined at 400 °C.: Bi 4f, Na 1s, K 2p, La 3d, Sr 3d, Ti 2p, O 1s.

**Figure 8:**
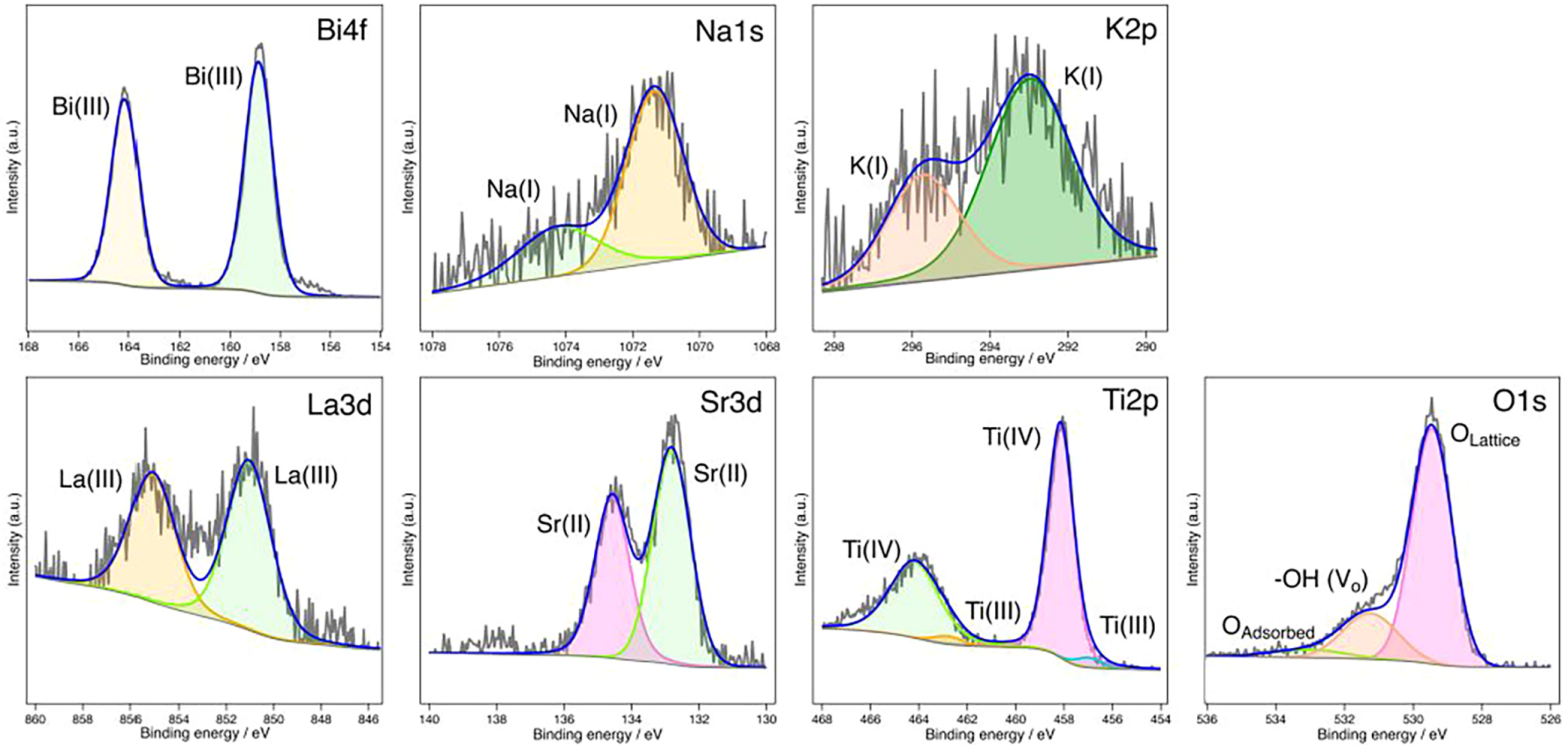
XPS spectra of BNKLST with F-127 synthesized at pH 9.0, calcined at 1000 °C: Bi 4f, Na 1s, K 2p, La 3d, Sr 3d, Ti 2p, O 1s.

Similarly, a tendency for a relative decrease in the Ti­(III) shoulder intensity in the Ti 2p spectrum was noticed for the sample thermally treated at 1000 °C, corroborating the oxidation of Ti­(III) to Ti­(IV) indicated by the TGA mass increase at approximately 910 °C. These results suggest that the concentration of oxygen-deficient species decreases with an increase in the calcination temperature. Moreover, the surface atomic proportions of the BNKLST samples were confirmed by XPS and are listed in Table 3, showing that the results are in good agreement with the nominal stoichiometry.

**Table 3:** Atomic Proportion of XPS Spectra of BNKLST with F-127 Synthesized at pH 9.0, Calcined at 400 and 1000 °C: Bi 4f, Na 1s, K 2p, La 3d, Sr 3d, Ti 2p, O 1s

sample	Bi 4f	Na 1s	K 2p	La 3d	Sr 3d	Ti 2p	O 1s
BNKLST 400 °C	4.47	4.49	4.02	3.98	4.51	19.27	59.26
BNKLST 1000 °C	4.33	3.91	4.26	3.98	4.22	19.75	59.55

### 3.6 Optical Properties

UV–vis DRS measurements were performed to investigate how these physicochemical properties affect light absorption characteristics. Figure 9 shows the UV–vis DRS of the samples synthesized with F-127 at pH 9.0 and subsequently calcined at 400 and 1000 °C. The sample calcined at 400 °C exhibited strong absorption primarily in the ultraviolet region below 400 nm but also showed a slight absorption shoulder extending into the visible light region, approximately between 400 and 500 nm. The absorption peak in the UV region can be assigned to a charge-transfer (CT) transition with a maximum at 322 nm, which is attributed to the transfer of electrons from Ti­(III) 3d orbitals to oxygen antibonding orbitals. The absorption shoulder in the visible region can be attributed to the d-d transitions due to the presence of Ti­(III).^46^ The existence of Ti­(III), which was also confirmed by XPS analysis, is a direct consequence of the formation of oxygen vacancies for charge compensation. Generally, oxygen vacancies in metal oxides form new localized electronic states within the bandgap.^47,48^ On the other hand, the spectrum of the sample calcined at 1000 °C showed an absorption edge at around 390–400 nm, with strong absorption predominantly in the UV region and negligible absorption in the visible light region. This optical absorption characteristic is consistent with that of typical wide-bandgap perovskite semiconductors like SrTiO_3_.^49^

**Figure 9:**
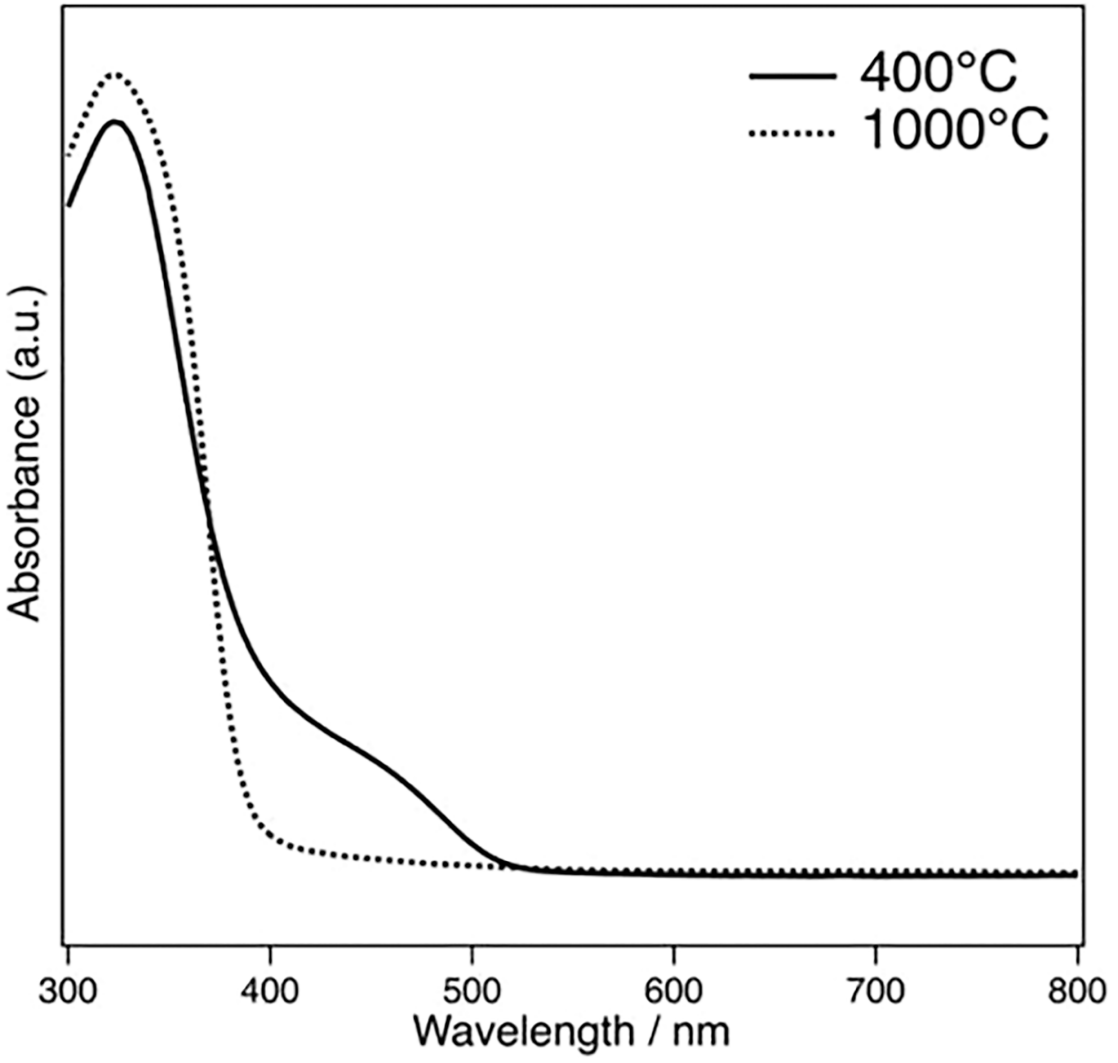
UV–vis DRS of BNKLST with F-127 synthesized at pH 9.0, calcined at 400 and 1000 °C.

### 3.7 Photocatalytic Activity

The photocatalytic activity of the prepared BNKLST samples was evaluated and reported here for the first time by the degradation of MB dye under UV irradiation (250–450 nm). Figure 10a shows the MB degradation curves by BNKLST samples synthesized with F-127 at pH 9.0 and calcined at different temperatures (400, 500, 600, and 1000 °C). Commercial SrTiO_3_ (Sigma-Aldrich, particle size <100 nm) was used as a reference material for comparison. The experiment involved stirring the mixture in the dark for 60 min to establish the adsorption–desorption equilibrium of MB on the nanoparticle surface, followed by UV irradiation. A blank experiment was also conducted under UV irradiation in the absence of the photocatalyst. The change in MB concentration in the dark indicated that the initial adsorption amount tended to be higher for samples with larger specific surface areas. The degradation of MB is often known to follow pseudo-first-order kinetics.^33^ Therefore, plots of *ln*(*C*_0_/*C*) vs irradiation time were plotted (Figure 10b), and the *k* of each sample is shown in Figures 10b,c. The sample calcined at 400 °C exhibited the highest *k* value (0.015 min^–1^), followed by the sample calcined at 1000 °C (0.010 min^–1^). These activities were higher than those of commercial SrTiO_3_ (0.0023 min^–1^). The high activity of the sample calcined at 400 °C can be attributed to its large specific surface area of 50.4 m^2^/g, as confirmed by the BET analysis. This provides an abundance of adsorption sites for MB molecules, increasing the reaction probability.^50^ Additionally, the influence of the phase associated with the aragonite group, confirmed by XRD, on the activity requires careful consideration. Previous research has reported that coexisting SrCO_3_ in SrTiO_3_-based systems can act as electron trapping sites, promoting charge separation, or potentially function as adsorption sites for reactants (such as O_2_), thereby enhancing reaction efficiency.^51–53^ However, in the current BNKLST system, the samples calcined at 500 °C (*k* = 0.0072 min^–1^) and 600 °C (*k* = 0.0093 min^–1^), where the aragonite-group phase was more evident in XRD, exhibited lower activity than that of the 400 °C sample. This fact suggests that, in this material system, the presence of the aragonite-group phase does not directly lead to enhanced activity, or that even if aragonite group had some positive effects (e.g., electron trapping or enhanced reactant adsorption), other factors such as a significant decrease in specific surface area and changes in crystallinity might have predominantly caused the overall reduction in activity. On the other hand, the sample calcined at 1000 °C also exhibited a high *k* value (0.010 min^–1^), despite its small specific surface area of 8.99 m^2^/g. For reference, the measured specific surface area of commercial SrTiO_3_ was 23.6 m^2^ g^–1^, suggesting that the high photocatalytic activity of the sample calcined at 1000 °C is not primarily governed by surface area effects. As indicated by XRD analysis, this sample is a single-phase perovskite and possesses high crystallinity. High crystallinity itself is a key factor that enhances photocatalytic reaction efficiency by suppressing photogenerated carrier recombination and improving charge carrier mobility.^54^ In addition, the presence of multiple A-site cations may also contribute to the activity by locally modifying the electronic structure and defect states, thereby promoting charge separation and facilitating carrier transport.^48^ Nevertheless, even when such high crystallinity is not achieved at low calcination temperatures, maintaining a large specific surface area through nanostructure control using sol–gel and soft-templating approaches remains an effective strategy for obtaining higher photocatalytic activity.

**Figure 10:**
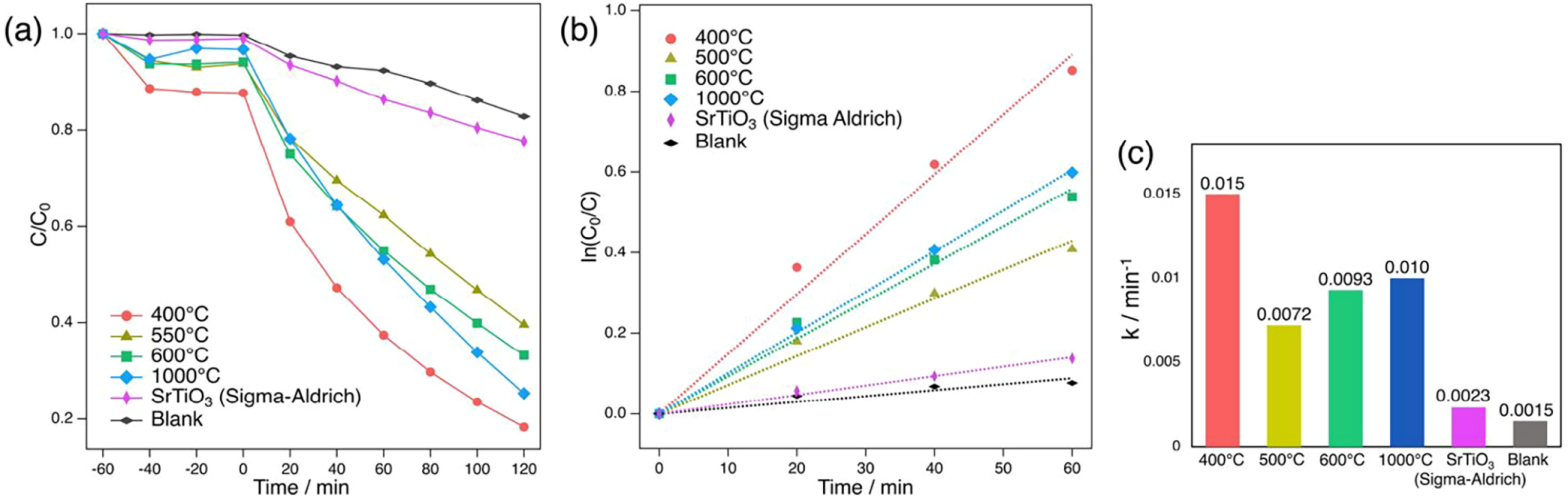
(a) Adsorption–desorption equilibrium (60 min in the dark) and subsequent photodegradation of MB, (b) corresponding first-order kinetic plots, and (c) apparent rate constant comparison.

## 4 Conclusions

In this study, we attempted to improve the structural properties and photocatalytic activity of the perovskite-type multicomponent metal oxide BNKLST by employing a sol–gel method alongside surfactant F-127 in its synthesis. As a primary achievement, the specific surface area of the material could be increased by utilizing F-127 as a template. Notably, for perovskite-type multicomponent metal oxides, which often require a high temperature of calcination at 1000 °C or above, we demonstrated that applying this approach enables samples calcined at a relatively low temperature of 400 °C to exhibit high photocatalytic activity. It is considered that while these low-temperature synthesized samples may not necessarily be single-phase and their crystallinity may not be perfect, the large specific surface area imparted by F-127 contributes to their remarkable activity. In addition, calcination at 1000 °C was confirmed to produce a highly crystalline, single-phase material, successfully forming a homogeneous HEO. The relatively good photocatalytic activity of this sample is attributed to its high crystallinity, which effectively suppresses the recombination of charge carriers.

## References

[1] Mohamadpour F., Amani A. M. (2024). Photocatalytic systems: reactions, mechanism, and applications. RSC Adv..

[2] Sohail M., Rauf S., Irfan M., Hayat A., Alghamdi M. M., El-Zahhar A. A., Ghernaout D., Al-Hadeethi Y., Lv W. (2024). Recent developments, advances and strategies in heterogeneous photocatalysts
for water splitting. Nanoscale Adv..

[3] Huang, R. ; Zhao, H. ; Chen, Z. High-entropy materials for photocatalysis Nano Materials Science 2024 10.1016/j.nanoms.2024.09.002.

[4] Akrami S., Edalati P., Fuji M., Edalati K. (2021). High-entropy ceramics:
Review of principles, production and applications. Mater. Sci. Eng. R Rep..

[5] Sun Y., Dai S. (2021). High-entropy materials for catalysis: A new frontier. Sci. Adv..

[6] Deng C., Wang T., Wu P., Zhu W., Dai S. (2024). High entropy
materials for catalysis: A critical review of fundamental concepts
and applications. Nano Energy.

[7] Han L., Zhu S., Rao Z., Scheu C., Ponge D., Ludwig A., Zhang H., Gutfleisch O., Hahn H., Li Z., Raabe D. (2024). Multifunctional
high-entropy materials. Nat.
Rev. Mater..

[8] Tsubota H., Jitianu A., Kawamura G. (2025). Recent advances in high-entropy oxides
for photocatalytic applications. ACS Mater.
Lett..

[9] Wang Y., Mi J., Wu Z.-S. (2022). Recent status and challenging perspective of high entropy
oxides for chemical catalysis. Chem. Catal..

[10] Güler Ö., Boyrazlı M., Albayrak M. G., Güler S. H., Ishihara T., Edalati K. (2024). Photocatalytic
hydrogen evolution
of TiZrNbHfTaO_x_ high-entropy oxide synthesized by mechano-thermal
method. Materials.

[11] Fu H., Li S., Lin Y., Wu X., Lin T., Zhao C., Gao M., Lin C. (2024). Enhancement
of piezo–photocatalytic activity
in perovskite (Bi_0.2_Na_0.2_Ba_0.2_K_0.2_La_0.2_)­TiO_3_ oxides via high entropy
induced lattice distortion and energy band reconfiguration. Ceram. Int..

[12] Akrami S., Murakami Y., Watanabe M., Ishihara T., Arita M., Fuji M., Edalati K. (2022). Defective
high-entropy oxide photocatalyst
with high activity for CO_2_ conversion. Appl. Catal., B.

[13] Maeda K. (2011). Photocatalytic
water splitting using semiconductor particles: History and recent
developments. J. Photochem. Photobiol. C: Photochem.
Rev..

[14] Poolakkandy R. R., Menamparambath M. M. (2020). Soft-template-assisted synthesis: a promising approach
for the fabrication of transition metal oxides. Nanoscale Adv..

[15] Kim K.-W., Park B., Kim J., Jo C., Kim J. K. (2023). Recent
progress in block copolymer soft-template-assisted synthesis of versatile
mesoporous materials for energy storage systems. J. Mater. Chem. A.

[16] Ashie M. D., Bastakoti B. P. (2024). Photocatalytic hydrogen evolution using mesoporous
honeycomb iron titanate. Small.

[17] Wang G., Qin J., Feng Y., Feng B., Yang S., Wang Z., Zhao Y., Wei J. (2020). Sol-gel synthesis
of spherical mesoporous
high-entropy oxides. ACS Appl. Mater. Interfaces.

[18] Einert M., Waheed A., Lauterbach S., Mellin M., Rohnke M., Wagner L. Q., Gallenberger J., Tian C., Smarsly B. M., Jaegermann W., Hess F., Schlaad H., Hofmann J. P. (2023). Sol-gel-derived
ordered mesoporous high entropy spinel ferrites and assessment of
their photoelectrochemical and electrocatalytic water splitting performance. Small.

[19] Rowell J. L., Kang M., Yoon D., Jiang K. Z., Jia Y., Abruña H. D., Muller D. A., Robinson R. D. (2024). Colloidal synthesis
of monodisperse high-entropy spinel oxide nanocrystals. J. Am. Chem. Soc..

[20] Wu K.-J., Tse E. C. M., Shang C., Guo Z. (2022). Nucleation and growth
in solution synthesis of nanostructures – From fundamentals
to advanced applications. Prog. Mater. Sci..

[21] Zhang J., Deng Y., Gu D., Wang S., She L., Che R., Wang Z.-S., Tu B., Xie S., Zhao D. (2011). Ligand-assisted
assembly approach to synthesize large-pore ordered mesoporous Titania
with thermally stable and crystalline framework. Adv. Energy Mater..

[22] Schüth F. (2001). Non-siliceous
mesostructured and mesoporous materials. Chem.
Mater..

[23] Yang P., Zhao D., Margolese D. I., Chmelka B. F., Stucky G. D. (1999). Block copolymer
templating syntheses of mesoporous metal oxides with large ordering
lengths and semicrystalline framework. Chem.
Mater..

[24] Wang Y., Liu J., Song Y., Yu J., Tian Y., Robson M. J., Wang J., Zhang Z., Lin X., Zhou G., Wang Z., Shen L., Zhao H., Grasso S., Ciucci F. (2023). High-entropy perovskites for energy
conversion and
storage: Design, synthesis, and potential applications. Small Methods.

[25] Biesuz M., Fu S., Dong J., Jiang A., Ke D., Xu Q., Zhu D., Bortolotti M., Reece M. J., Hu C., Grasso S. (2019). High entropy
Sr­((Zr_0.94_Y_0.06_)_0.2_Sn_0.2_Ti_0.2_Hf_0.2_Mn_0.2_)­O_3‑x_ perovskite synthesis by reactive spark plasma sintering. J. Asian Ceram. Soc..

[26] Jiang S., Hu T., Gild J., Zhou N., Nie J., Qin M., Harrington T., Vecchio K., Luo J. (2018). A new class
of high-entropy
perovskite oxides. Scr. Mater..

[27] Avcıoglu C., Avcıoglu S., Bekheet M. F., Gurlo A. (2023). Photocatalytic overall
water splitting by SrTiO_3_: Progress report and design strategies. ACS Appl. Energy Mater..

[28] Yang W., Zheng G. (2022). High energy storage
density and efficiency in nanostructured (Bi_0.2_Na_0.2_K_0.2_La_0.2_Sr_0.2_)­TiO_3_ high-entropy
ceramics. J.
Am. Ceram. Soc..

[29] Li H., Lai C., Wei Z., Zhou X., Liu S., Qin L., Yi H., Fu Y., Li L., Zhang M., Xu F., Yan H., Xu M., Ma D., Li Y. (2023). Strategies for improving
the stability of perovskite for photocatalysis: A review of recent
progress. Chemosphere.

[30] Lyu H., Hisatomi T., Goto Y., Yoshida M., Higashi T., Katayama M., Takata T., Minegishi T., Nishiyama H., Yamada T., Sakata Y., Asakura K., Domen K. (2019). An Al-doped SrTiO_3_ photocatalyst
maintaining sunlight-driven
overall water splitting activity for over 1000 h of constant illumination. Chem. Sci..

[31] Li Z.-X., Shi F.-B., Ding Y., Zhang T., Yan C.-H. (2011). Facile
synthesis of highly ordered mesoporous ZnTiO_3_ with crystalline
walls by self-adjusting method. Langmuir.

[32] Muniz F. T. L., Miranda M. A. R., Morilla
Dos Santos C., Sasaki J. M. (2016). The Scherrer equation and the dynamical
theory of X-ray
diffraction. Acta Crystallogr. A Found. Adv..

[33] Azeez F., Al-Hetlani E., Arafa M., Abdelmonem Y., Nazeer A. A., Amin M. O., Madkour M. (2018). The effect of surface
charge on photocatalytic degradation of methylene blue dye using chargeable
titania nanoparticles. Sci. Rep..

[34] Sarkar A., Djenadic R., Wang D., Hein C., Kautenburger R., Clemens O., Hahn H. (2018). Rare earth and transition
metal based
entropy stabilised perovskite type oxides. J.
Eur. Ceram. Soc..

[35] Yeh J. W., Chen Y. L., Lin S. J., Chen S. K. (2007). High-entropy alloys
– A New Era of exploitation. Mater. Sci.
For..

[36] Klobes, P. ; Meyer, K. ; Munro, R. G. Porosity and Specific Surface Area Measurements for Solid Materials. In NIST Special Publication 960–17; National Institute of Standards and Technology: Gaithersburg, MD, 2006; pp 24–26.

[37] Belsky A., Hellenbrandt M., Karen V. L., Luksch P. (2002). New developments in
the Inorganic Crystal Structure Database (ICSD): accessibility in
support of materials research and design. Acta
Crystallogr. B.

[38] Fuentes R. O., Woollins J. D., Baker R. T. (2010). Temperature effects
on structural
properties in the synthesis of nanocrystalline Zr_0.5_Ce_0.5_O_2_ solid solution: A study by XRD and HRTEM. J. Alloys Compd..

[39] Naikoo G. A., Thomas M., Anis Ganaie M., Sheikh M. U. D., Bano M., Hassan I. U., Khan F. (2016). Hierarchically
macroporous silver
monoliths using Pluronic F127: Facile synthesis, characterization
and its application as an efficient biomaterial for pathogens. J. Saudi Chem. Soc..

[40] Zhang W. X., Li L. Q., Li P. (2018). Thermal behavior and
characteristic
of submicron sized barium titanate ceramic synthesized by acetylacetonate
precursor. Ceram. Int..

[41] Li J., Li R., Wang W., Lan K., Zhao D. (2024). Ordered mesoporous
crystalline frameworks toward promising energy applications. Adv. Mater..

[42] Sun H., Dong C., Huang A., Zhan H., Wang G., Liu W., Ma B., Wang W. (2022). Transition metal doping induces Ti^3+^ to
promote the performance of SrTiO_3_ @TiO_2_ visible
light photocatalytic reduction of CO2 to prepare
C1 product. Chemistry.

[43] Shi X., Dai W., Li X., Yu Y., Zhu Z., Cui Z., Dong X. (2024). Lattice strain in high entropy oxides promote CO_2_ photomethanation. Small Methods.

[44] Wang Y., Yang X., Hou C., Yin F., Wang G., Zhu X., Jiang G., Li C. (2021). Improved catalytic activity and stability
of Ba substituted SrTiO_3_ perovskite for oxidative coupling
of methane. ChemCatChem.

[45] Nguyen T. X., Liao Y.-C., Lin C.-C., Su Y.-H., Ting J.-M. (2021). Advanced
high entropy perovskite oxide electrocatalyst for oxygen evolution
reaction. Adv. Funct. Mater..

[46] Lever, A. B. P. Inorganic electronic spectroscopy; Elsevier Science, 1984.

[47] Chatzitakis A., Sartori S. (2019). Recent advances
in the use of black TiO2 for production
of hydrogen and other solar fuels. ChemPhysChem.

[48] Ling H., Sun H., Lu L., Zhang J., Liao L., Wang J., Zhang X., Lan Y., Li R., Lu W., Cai L., Bai X., Wang W. (2024). Sustainable photocatalytic hydrogen
peroxide production over octonary high-entropy oxide. Nat. Commun..

[49] Takata T., Jiang J., Sakata Y., Nakabayashi M., Shibata N., Nandal V., Seki K., Hisatomi T., Domen K. (2020). Photocatalytic water splitting with
a quantum efficiency of almost
unity. Nature.

[50] Kudo A., Miseki Y. (2009). Heterogeneous photocatalyst
materials for water splitting. Chem. Soc. Rev..

[51] Jin S., Dong G., Luo J., Ma F., Wang C. (2018). Improved photocatalytic
NO removal activity of SrTiO_3_ by using SrCO_3_ as a new co-catalyst. Appl. Catal., B.

[52] Boga B., Moustakas N. G., Han Y., Jiao H., Kreyenschulte C., Naliwajko P., Duong T. T. H., Ding S., Ngo A. B., Hezam A., Peppel T., Cristea V.-M., Steinfeldt N., Strunk J. (2024). Design of SrTiO_3_-based catalysts for photocatalytic
CO_2_ reduction. Catal. Sci. Technol..

[53] Deng Y., Shu S., Fang N., Wang R., Chu Y., Liu Z., Cen W. (2023). One-pot synthesis
of SrTiO_3_-SrCO_3_ heterojunction
with strong interfacial electronic interaction as a novel photocatalyst
for water splitting to generate H_2_. Chin. Chem. Lett..

[54] Zhang Q. P., Xu X. N., Liu Y. T., Xu M., Deng S. H., Chen Y., Yuan H., Yu F., Huang Y., Zhao K., Xu S., Xiong G. (2017). A feasible
strategy
to balance the crystallinity and specific surface area of metal oxide
nanocrystals. Sci. Rep..

